# Asymmetrical monitoring of subjective asynchronies: a metacognitive generalized STEARC effect

**DOI:** 10.1007/s00426-025-02123-2

**Published:** 2025-04-28

**Authors:** Tutku Öztel, Martin Wiener, Fuat Balcı

**Affiliations:** 1 Department of Psychology, Koç University, Istanbul, Turkey; 2 Department of Psychology, George Mason University, Fairfax, Virginia, USA; 3 Department of Biological Sciences, University of Manitoba, 50 Sifton Road, Winnipeg, MB R3T 2M5 Canada

## Abstract

Previous studies have demonstrated that human participants can keep track of the magnitude and direction of their trial-to-trial errors in temporal, spatial, and numerical estimates, collectively referred to as “metric error monitoring.” These studies investigated metric error monitoring in an explicit timing/counting context. However, many of our judgments may also depend on temporal mismatches between stimuli where the temporal information is not processed explicitly, which eventually brings about the simultaneity perception. We investigated whether participants can monitor errors in their simultaneity perception. We tested participants in temporal orer judgment (TOJ) task, where they judged which of the two consecutive stimuli (one on each side of the screen) appeared first and reported their confidence rating for each TOJ. The results of all four experiments showed that the confidence judgements for correct judgments increased and for incorrect judgments decreased with longer absolute SOA. A more granular analysis showed that participants could only monitor their errors for left-first and bottom-first judgments, which suggests a metacognitive spatial–temporal association of response codes (STEARC) effect.

**Supplementary Information:**

The online version contains supplementary material available at 10.1007/s00426-025-02123-2.

## Introduction

Metacognition is “cognition about cognition” (Flavell, 1979). This higher-order ability is related to various cognitive processes such as decision-making, behavioral adaptation (Brown, 1987), and learning (Schraw et al., 2006). One of the most prominent aspects of metacognition stems from one’s ability to report their own errors/performance correctly, referred to as error/performance monitoring (Yeung & Summerfield, 2014). Although this ability was observed in many domains, including interval timing, no study has investigated whether humans can monitor the accuracy of their simultaneity judgments when interrogated as a function of relative temporal latencies (i.e., subjective asynchronies) between two stimuli.

The simultaneity perception is typically investigated with the temporal order judgments (TOJ), where participants report which of the two stimuli was presented first (Kostaki & Vatakis, 2018). Thus, instead of explicitly timed, TOJs are inferred from the arrival latencies (due to delays in reaching sensory organs and their neural transduction/processing) of stimuli (Sternberg & Knoll, 1973; also see Kostaki & Vatakis, 2018). This brings about distinctive cognitive processing concerning performed timing behaviors with motor input, such as reproducing a target time by demarcating elapsed time (i.e., temporal reproduction). We investigated whether humans can keep track of the accuracy of their simultaneity perception with respect to the subjective asynchronies of two stimuli.

### Metric information processing as part of metacognition

The error monitoring aspect of metacognition is keeping track of an agent’s errors without requiring external feedback. In its explicit form, error monitoring is operationalized by the overall match between the objective accuracy and the subjective confidence rating regarding the accuracy of the decision (e.g., “How confident are you that your response is correct?”; for a detailed discussion, see Fleming, 2014). Error monitoring performance has mostly been investigated in perceptual decision-making and memory domains. Overall, these studies showed that humans can adjust their relative confidence according to the decision accuracy in both perceptual (e.g., Fleming & Dolan, 2012b, 2012c; also see Fleming et al., 2012a) and memory domains (e.g., Vandenbroucke et al., 2014) but these domains limit the nature of error monitoring to only categorical judgments that can be either correct or incorrect (categorical errors). However, in many real-life situations, judgments can deviate from an objective target at different degrees and in different directions, attributing explicit metric properties to errors (as opposed to performance that is merely binary in its psychometric nature). For example, one can be late or early (direction) for an appointment by 5–45 min (magnitude). Thus, a more comprehensive, complete, and generalizable investigation of error monitoring requires considering the explicit metric, in addition to the categorical, characteristics in error monitoring studies, which captures both continuous (in the form of magnitude) and categorical (in the form of relative direction) features of the decisions made in many daily life situations^1^.

To this end, recent studies investigated the error monitoring abilities in metric domains such as time, space, and number. For instance, Akdoğan and Balcı (2017) tested participants in a temporal reproduction task where participants reproduced a given target duration by button presses. After each reproduction, participants reported their subjective confidence regarding the proximity of their reproduction to the given target duration. Finally, participants were asked to report whether their reproduction was shorter or longer than the target duration. They showed that participants could correctly report the magnitude and direction of their temporal errors (aka temporal error monitoring). Crucially, this ability has been replicated many times in temporal (e.g., Öztel & Balcı, 2021, Öztel & Balcı, 2023a; 2023bb) and other magnitude domains such as space (e.g., line length estimations as in Duyan & Balcı, 2020; orientation estimations as in Bertana et al., 2021; see also Recht et al., 2021) and number (e.g., Duyan & Balcı, 2018, 2019), pointing to the domain-general characteristics of this ability (for a detailed discussion see Yallak & Balcı, 2021). These results suggest an extended scope of metacognitive abilities that can also take metric forms (“metric error monitoring ability” - for a detailed discussion, see Öztel & Balcı, 2024).

With their current methodological approach, metric error monitoring studies capture the metric characteristics of errors associated only with the explicit prospective timing of a single stimulus, which typically requires a motor output. One recent study contrasted the temporal error monitoring abilities across performed vs. observed errors in the temporal reproduction task and found that the agency manipulation affected the two aspects of temporal error monitoring differentially (i.e., confidence and error directionality judgments - Oztel & Balci, 2024). However, no study has investigated how relative subjective asynchronies (perceived passively instead of explicitly as in earlier tasks) are involved in error monitoring processes of simultaneity perception.

One methodological approach to quantify subjective asynchronies is via the TOJ task. In this task, participants are demonstrated asynchronously presented two stimuli with different stimulus onset asynchronies (SOA) in the subsecond range and asked to report which stimulus appeared first (e.g., “left” or “right” stimulus). The median value of the psychometric function fitted to the response probabilities corresponds to the point of subjective simultaneity (PSS), which refers to the time point at which participants perceive two stimuli as simultaneously presented (for a detailed discussion, see Kostaki & Vatakis, 2018). Thus, subjective asynchrony can be quantified between the experimental SOA (an index of objective simultaneity of two stimuli) and the PSS.

These methodological characteristics can also reflect itself in cognitive forms, imposed by the fact that TOJ and reproduction tasks capture different interval ranges (“automatic timing” that concerns subsecond intervals as in TOJ and “cognitively controlled timing” that concerns supra-second intervals as in reproduction; Buhusi & Meck, 2005). As a result, the inference of TOJ from the passive perceptual processing of stimuli (e.g., as opposed to explicit timing) renders it a relatively instantaneous operation compared to temporal reproductions. The neural counterparts of this cognitive difference have also been documented. For subsecond timing (automatic timing), supplementary motor area (SMA), primary motor cortex, and primary sensory cortex are implicated, while for suprasecond timing (cognitively controlled timing), dorsolateral prefrontal cortex (dLPFC), intraparietal sulcus, premotor cortex are implicated (Buhusi & Meck, 2005). Due to these critical differences, the results obtained from the earlier temporal error monitoring studies cannot be generalized to error monitoring dynamics in simultaneity perception. This shortcoming highlights a crucial gap in the current literature on metric error monitoring.

Reporting confidence in TOJ tasks has been occasionally documented in the literature (e.g., Faivre et al., 2020; Craig, 2005). For example, in a recent study, Faivre and colleagues (2020) tested participants in a TOJ task to investigate the effect of sensorimotor conflict on metacognitive performance. After each judgment, participants rated their confidence level regarding the accuracy of their responses. However, they did not investigate error monitoring of the metric characteristics of the TOJ. In another study, Craig (2005) investigated the trajectory effects on the TOJ, such that the direction of the movement could change the TOJ in three experiments. The first two experiments pointed out that the trajectory effect is especially pronounced for the cases where the SOA is relatively short. Craig (2005) asked participants to report their confidence level in the final experiment to test whether subjective confidence judgments might drive this effect. However, these authors did not investigate the metacognitive aspects of TOJ, either. Finally, Keane and colleagues (2020) investigated the confidence judgments in TOJ concerning the effect of rapid temporal recalibration (i.e., the effect of two stimuli to be perceived as appearing simultaneously after a brief asynchronous presentation of them). They found a negative recalibration in the form of a general decrease in confidence judgments as a function of repeated same-SOA consecution (Keane et al., 2020). Still, none of these studies investigated the error monitoring dynamics in simultaneity perception.

### Current study

In four experiments, the current study investigated whether humans can monitor errors in their simultaneity perception as a function of subjective asynchronies, which has not been addressed in previous studies. Participants were tested in TOJ tasks where they reported which of the two visual stimuli appeared first (left or right in the first three experiments and top or bottom in the last experiment). After each response, participants reported their subjective confidence regarding the accuracy of their judgment. If the participants could monitor their simultaneity perceptions, a) their confidence ratings should increase and decrease as a function of absolute SOAs for correct and incorrect responses, respectively (due to higher discriminability with increased SOA, which is also associated with accuracy (e.g., Kepecs et al., 2008; Sanders et al., 2016). Additionally, if the simultaneity monitoring depends on subjective asynchronies, participants’ confidence ratings should be modulated by the match between their TOJ and subjective simultaneity. Furthermore, potential asymmetries in metacognitive abilities across the response codes could indicate a metacognitive spatial-temporal association of response codes (STEARC) effect, which refers to faster left-mapped responses for smaller magnitudes (e.g., Ishihara et al., 2008; Dehaene et al., 1993; Gevers et al., 2004; for a detailed review see Bonato et al., 2012). This potential metacognitive asymmetry will also be tested in the current study. Finally, if participants cannot monitor their simultaneity perception, the confidence judgements should not be reliably predicted by the subjective asynchronies, regardless of the accuracy and response type (i.e., “left/right first” as in the first three experiments or “bottom/top first” as in the fourth experiment).

## Experiment 1

### Method

#### Participants

Twenty five undergraduate students from Koç University participated in this experiment for half extra course credit. Seven participants were excluded from the formal analyses for having an R^2^ < 0.80 for psychometric function fits. Twelve of the remaining participants were female (*M*_age_ = 20.61, *SD*_age_ = 1.46), and one was left-handed. All participants were native Turkish speakers, had normal or corrected vision, and did not use any psychiatric drugs. All participants provided informed consent before the experiment.

#### Materials and procedure

The experiment was conducted in a testing room on a standard Dell PC (60 Hz screen refresh rate, 23.8” Dell P2414H monitor, ~ 16.66 milliseconds interframe interval). Each trial began with a fixation cross appearing on the center of the screen for 30 frames (i.e., 500 ms). Two white discs appeared consecutively on each side of the fixation cross with different stimulus onset asynchronies (SOA). Consequently, one of the discs appeared before the other disc. The SOA between the two discs ranged between 1 (~ 16.66 milliseconds) and 12 frames (~ 200 milliseconds). Participants were asked to fixate on the fixation cross as they performed the task. Upon the disappearance of both discs, participants were asked to report which stimuli appeared first by pressing the left or right arrow on the keyboard for “left stimulus appeared first” and “right stimulus appeared first” responses, respectively. Whether the left vs. right stimulus appeared first was randomized across experimental trials with equal probabilities. However, the probability of left vs. right stimulus’ first appearance was not equal across different SOAs. After each response, participants reported how confident they were regarding the accuracy of their responses by pressing 1 (low confidence), 2 (medium confidence), or 3 (high confidence) buttons on the keyboard. Participants did not receive any feedback regarding the accuracy of their responses throughout the experiment.

**Fig. 1:**
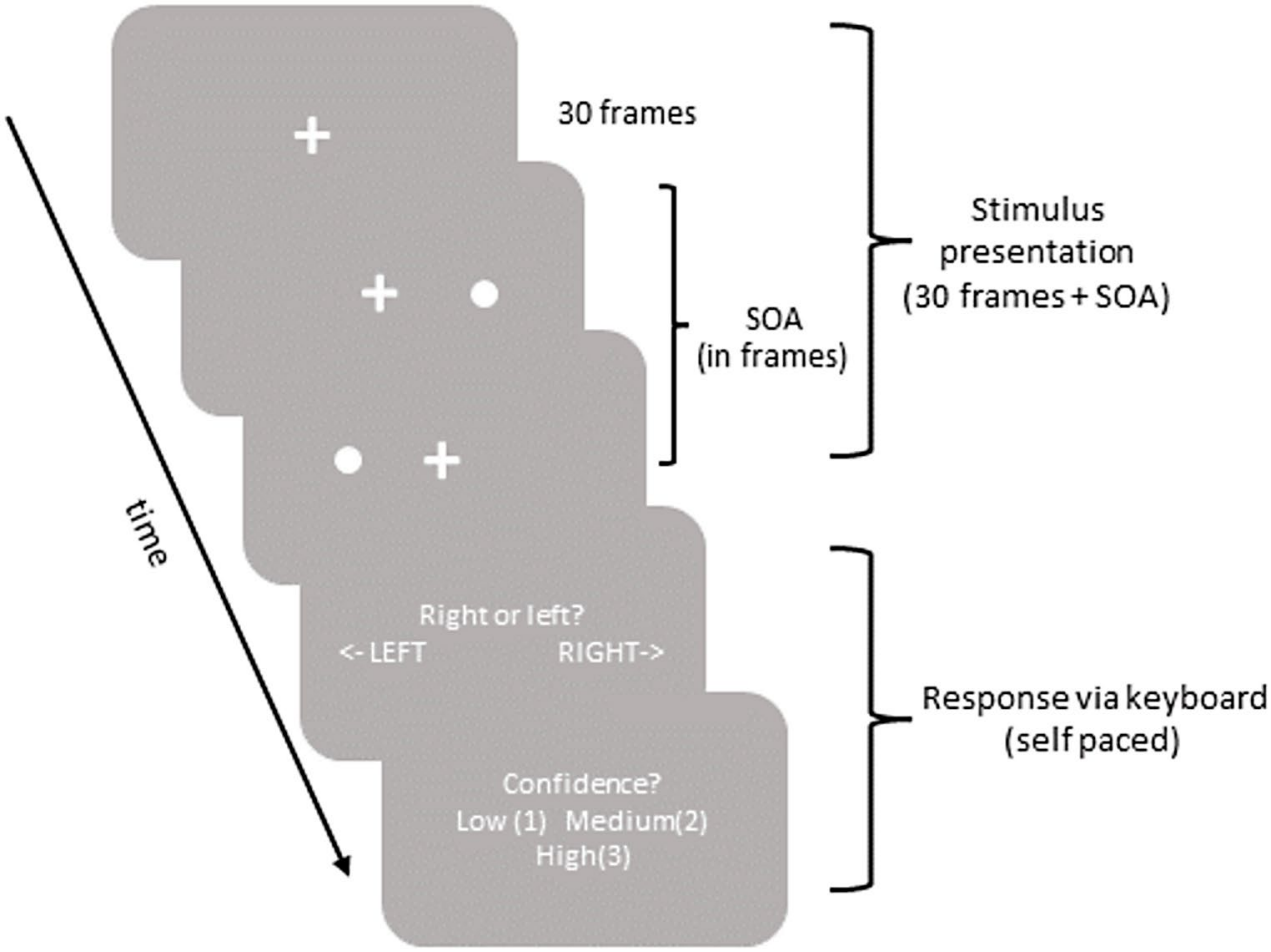
An example trial sequence for the “right first” condition

The experiment consisted of five separate test blocks separated by 1 min long mandatory breaks. Each test block consisted of 8 consecutive SOAs incrementing one frame to the maximum SOA of the nearest block (e.g., SOAs in block 1: 1–8 frames (i.e., ~ 16.66–133.28 milliseconds); SOAs in block 2: 2–9 frames (i.e., ~ 33.32–150 milliseconds). Each SOA was tested five times within a test block, which resulted in 40 trials per test block (i.e., 8 SOAs x 5). As a result, the experiment consisted of 200 trials in total (i.e., 40 trials x 5 blocks). The order of the SOAs was randomized across the blocks, and the order of the blocks was randomized across participants. Before the experiment, participants completed eight practice trials to familiarize themselves with the task (SOA range: 3–10 frames (i.e., ~ 50–166.6 milliseconds). The experiment took approximately 15 min to complete. All stimuli presentation and response recordings were done in the Builder mode of the *PsychoPy* experimentation platform (v2022.2.0, Peirce et al., 2019). All responses were recorded via a mechanical keyboard (Zalman, ZM-K500). Figure 1 illustrates one trial sequence in the experiment.

All data and codes used for this study are available in the Open Science Framework (OSF) repository (https://osf.io/sw6cq/?view_only=105ad3a7fa844f7cbaacb55ae97ef066). This study was not preregistered. The local institutional review board (IRB, protocol number: 2017.215.IRB3.118) approved all the procedures.

### Analytical approach

#### Psychometric functions

We first re-coded the SOAs in a way that captured both the SOA between the two discs and the side condition. To this end, the composite SOA ranged between 1 and 24 (1: right disc appeared twelve frames before the left disc, 24: left disc appeared twelve frames before the right disc).

**Fig. 2:**
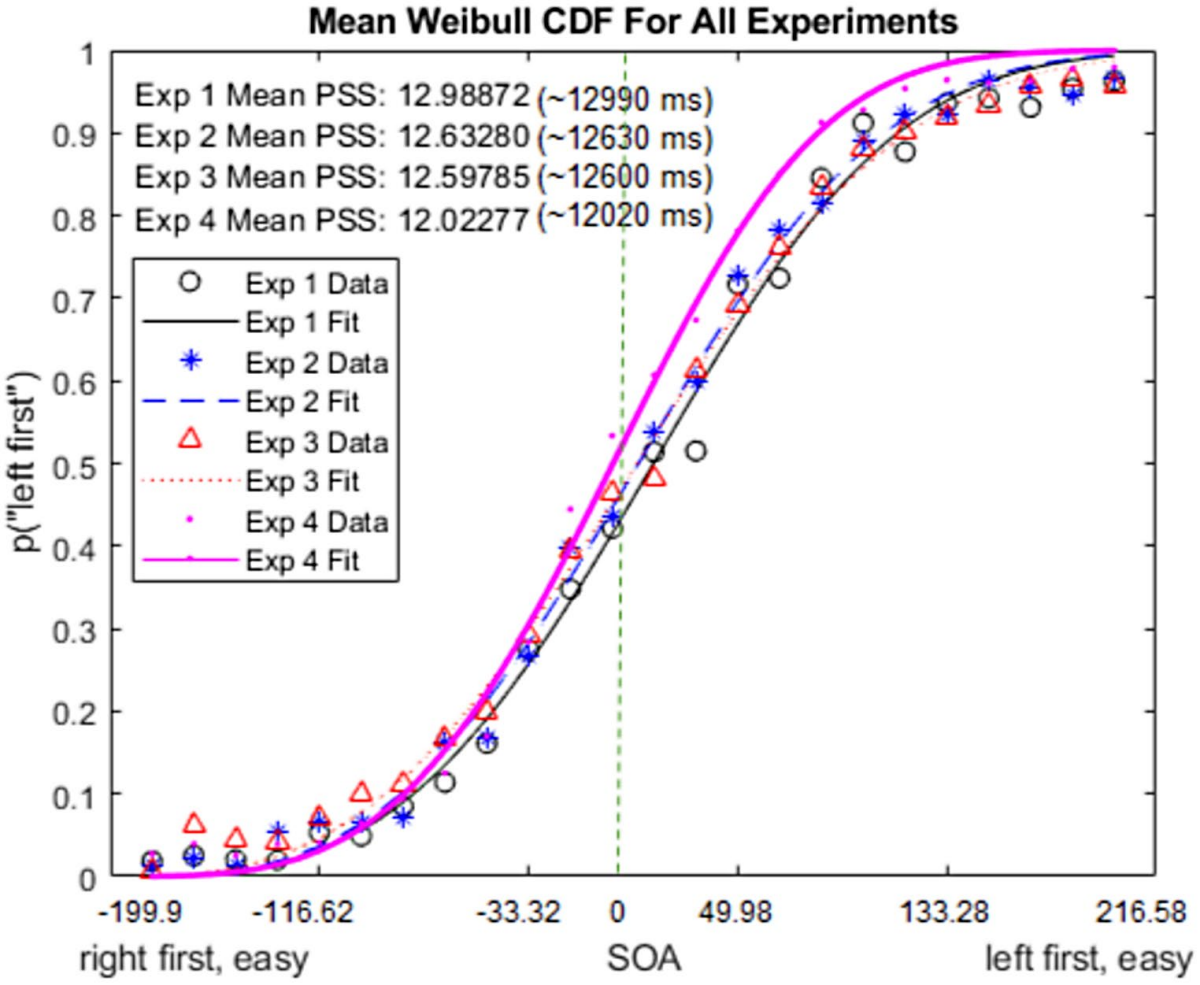
Mean Weibull cumulative density function across participants for three experiments. The vertical dashed line depicts the objective simultaneity (12 frames). The SOAs are converted in milliseconds unit

We fit the standard Weibull cumulative distribution function (cdf - Matlab 2023b) to the probability of “long first” responses as a function of SOA. We calculated the point of subjective simultaneity (PSS) as the median of the Weibull cdf (1- *e*^−(*x*/*λ*)^*k*^ where λ and k depict the scale and shape parameter, respectively). The PSS then corresponds to the inverse of the function evaluated at 0.5 (i.e., the median of the function): PSS = λ(ln2)^1/k^. Figure 2 demonstrates the mean Weibull cdf fits across participants for four experiments.

#### Quantifying subjective asynchrony

The PSS corresponds to the number of frames the participant perceives that the two stimuli appeared simultaneously. Accordingly, a positive PSS depicts bias towards perceiving the left disc onset earlier. To quantify the subjective asynchrony, the SOAs were then recoded per participant by subtracting the corresponding participant’s PSS from the SOAs (PSS-centered SOA). As a result, the PSS-centered SOAs that are larger than zero would indicate the subjective “left first” side of the centered SOA continuum. Similarly, PSS-centered SOAs smaller than zero would indicate the subjective “right first” side of the continuum. *2.2.3. PSS-centered accuracy (“subjective accuracy”).* In line with the previous description, we coded all “right first” responses for negative PSS-normalized SOA (subjective “right first” side of the continuum) as “correct.” Similarly, we coded all “left first” responses with positive PSS-centered SOA as “correct” (subjective “left first” side of the continuum).

#### Linear mixed effects analyses

To capture the whole data in a single model, we tested our hypotheses in a linear mixed effects model, which is especially useful for the within-subject designs where the independent observations assumption of the ordinary least squares (OLS) analysis is violated. We used the restricted maximum likelihood method (REML) for the parameter estimations, and for the degrees of freedom (df), we used the Satterthwaite method. For all models, we centered the continuous predictors across the participants. We ran the linear mixed effects analyses in Jamovi (version 2.4.11.0; The jamovi project, 2019; R Core Team, 2018) using the GAMLj package (Gallucci, 2019).

In all experiments, we ran two different models. In Model 1, we aimed to investigate whether the participants’ confidence ratings were sensitive to absolute PSS-centered SOA. To this end, we included the absolute value of PSS-centered SOA and the PSS-centered accuracy (will be hereafter referred to as “accuracy” for simplicity) in the model. We predicted the overall confidence for correct responses to increase and for incorrect responses to decrease with longer PSS-centered SOAs. We tested our hypothesis with the following model: Confidence Judgements ~ |PSS-centered SOA| * Accuracy + (1|participant) [Model 1].

While Model 1 can capture the overall ability to monitor simultaneity judgment, it cannot address whether this ability can be observed similarly for different response options. To verify the robustness of the simultaneity monitoring across response options, we also tested whether participants could monitor errors in their judgments regarding directional judgments. Accordingly, the confidence judgements should be maximal for the extreme ends of SOA (i.e., high confidence for too small and too large PSS-centered SOAs) due to lower relative difficulty of onset discrimination. Crucially, this relationship should exhibit different directions for “left first” and “right first” responses, which can be addressed with an interaction term that investigates the slope difference between the two responses. Thus, we predicted that the confidence judgments should increase for left-first responses and decrease for right-first responses as a function of PSS-centered SOAs (i.e., a significant interaction between PSS-centered SOA and response; also note that negative PSS-centered SOAs correspond to “right-first” SOAs).

We tested our hypothesis with the following model: Confidence Judgement ~ PSS-centered SOA* Response + (1|participant) [Model 2].

In both models described above, “~” stands for predicted from, and “(1|participant)” stands for “random intercept across participants.” All models included all lower terms. Accordingly, Model 2 can reveal potential differences in the response option characteristics while eliciting metacognitive monitoring in simultaneity perception.

To avoid overly complex models, we kept the random effect structure as the simplest that could test our hypotheses. Note that we fitted the same models with the most complex random effect structure possible. All these models either (a) resulted in convergence or overfitting issues or (b) revealed similar results to those we reported. Thus, our analytical approach does not yield a change in the conclusions drawn from them. Furthermore, we also analyzed the data, treating confidence ratings as continuous and ordinal variables. In both approaches results yielded the same coefficients and Bayesian Information Criterion (BIC) scores.

### Results

#### Response bias

We first investigated whether there was an overall bias in the accuracy favoring one type of side response (i.e., overall higher accuracy for “left” or “right first” responses) or an overall bias in the side responses (i.e., overall higher rate of either “left” or “right first” responding). Two separate paired sample t-tests revealed no bias in the side responses favoring one response type (*t*(17) = 1.97, *p* = 0.065, Cohen’s d = 0.47; BF_10_ = 1.18, % error = 2.378e-4: where data are only 1.18 times more likely under the alternative than the null hypothesis); which also applied for the response accuracy (*t*(17) = 1.92, *p* = 0.072, Cohen’s d = 0.45; BF_10_ = 1.089, % error = 2.374e-4; where data are only 1.089 times more likely under the alternative than the null hypothesis). These results show no overall bias across side responses that could influence the models we tested for our main hypotheses.

#### Model 1

**Fig. 3:**
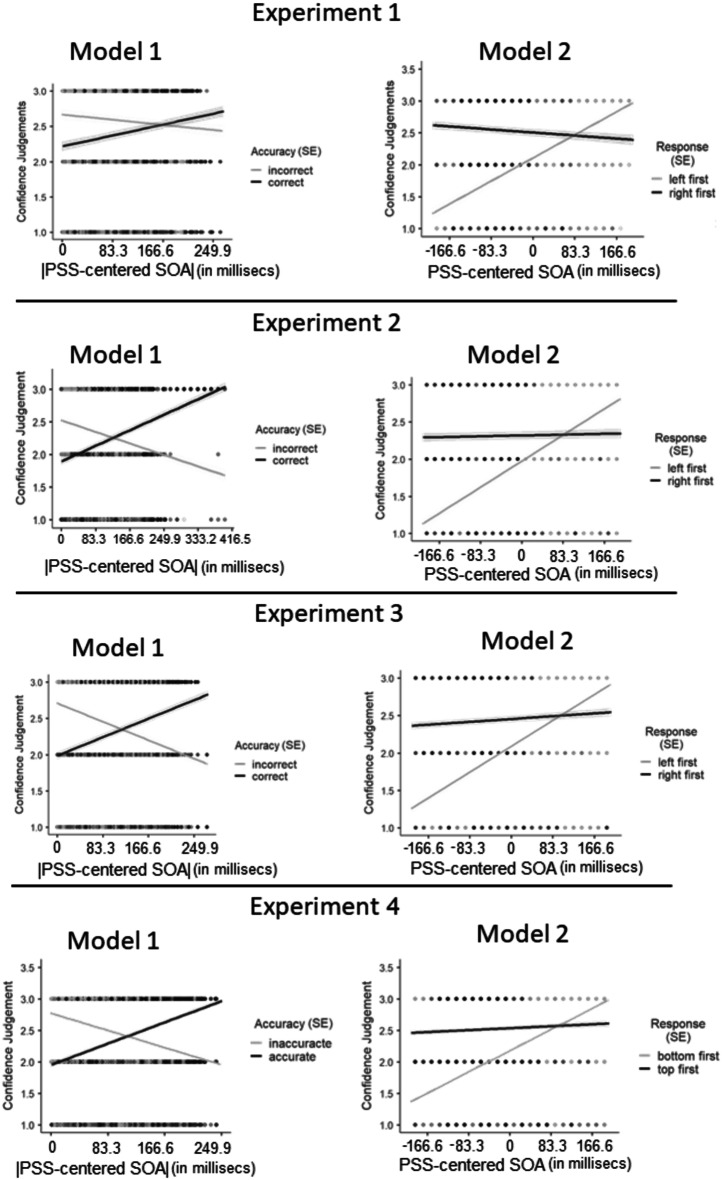
The relationship between confidence judgements and absolute PSS-centered SOA separately for correct and incorrect responses (left column) and the relationship between confidence judgments and PSS-centered SOAs (in milliseconds unit) separately for left-first and right-first responses (right column). Each row depicts three experiments. Grey-shaded areas depict the standard error of the estimates (SE). The negative values on the x-axis depict the onset of the stimulus appearing first on the right side (Experiment 1-3) and bottom of the screen (Experiment 4)

Although the relationship between the absolute PSS-centered SOA and confidence was significantly positive for correct judgments (ß_correct_ = 0.032, *SE* = 0.005, 95% CI = [0.022 0.042], *p* < 0.001), this relation was significantly negative for the incorrect responses (ß_incorrect_ = -0.016, *SE* = 0.006, 95% CI = [-0.027 -0.0046], *p* < 0.001). By transitivity, the difference between the slopes was also statistically significant (ß_incorrect−correct_ = -0.05, *SE* = 0.008, 95% CI = [-0.063 -0.032], *p* < 0.001). The first row of the left panel in Fig. 3 illustrates the relationship between absolute PSS-centered SOA and confidence judgements separately for correct and incorrect judgments in Experiment 1 (SOM S4 for binned version). Surprisingly, Model 1 also revealed that overall, participants reported higher confidence when their judgments were inaccurate (*M*_incorrect−correct_ = 0.17, *SE* = 0.024, 95% CI = [0.13 0.22], *p* < 0.001). This result, however, could be an artifact of our coding scheme for accuracy (as described in “PSS-centered accuracy”), instead of representing a genuine cognitive reflection (see Raw data - model).

#### Model 2

For Model 2, results revealed a confidence bias by showing significantly higher confidence for right first responses (*M*_right first − left first_ = 0.40, *SE* = 0.031, 95% CI = [0.34 0.46], *p* < 0.001). Simple slopes pointed to a significant positive slope for the left first responses (ß_left first_ = 0.07, *SE* = 0.0031, 95% CI = [0.066 0.078], *p* < 0.001). However, albeit very low in value, the slope was negative for the right-first responses (ß_right first_ = -0.010, *SE* = 0.0036, 95% CI = [-0.017 -0.0026], *p* = 0.007). By transitivity, the slope difference was also statistically significant (ß_right first − left first_ = -0.082, *SE* = 0.005, 95% CI = [-0.091 -0.073], *p* < 0.001). The first row of the right panel in Fig. 3 illustrates the relationship between PSS-centered SOA and confidence judgements separately for different order judgments for Experiment 1.

#### Raw data - model

To capture whether the simultaneity monitoring depends exclusively on the subjective asynchronies, we also investigated our research question in a raw data-model in which we only included the untransformed data as predictors. Accordingly, the linear mixed effects model was as follows:

$$\begin{gathered}{\text{Confidence judgements} \sim {Objective\:accuracy }} \hfill \\{\text{* SOA + 1|participants}} \hfill \\ \end{gathered} $$

For Experiment 1, the model revealed a mean difference between correct and incorrect responses in terms of confidence judgments (*M*_correct − incorrect_ = 0.44, *SE* = 0.07, 95% CI = [-0.57 -0.31], *p* < 0.001). This result further validates that the unexpected higher confidence for inaccurate responses in Model 2 could be a coding artifact instead of reflecting cognitive processing. Further, the overall effect of SOA on confidence judgments was statistically significant (ß = 0.099, *SE* = 0.006, 95% CI = [0.087 0.11], *p* < 0.001). The simple slopes revealed a significantly positive slope for both correct and incorrect responses (ß_incorrect_ = 0.091, *SE* = 0.012, 95% CI = [0.069 0.11], *p* < 0.001; ß_correct_ = 0.11, *SE* = 0.004, 95% CI = [0.097 0.11], *p* < 0.001). However, the slope was not different between correct and incorrect responses (ß_correct − incorrect_ = 0.014, *SE* = 0.012, 95% CI = [-0.039 0.010], *p* = 0.24). Together, these results reveal that participants cannot discriminate their (objective) inaccurate responses from the (objective) accurate ones.

### Interim discussion

Our results revealed a positive and negative linear relationship between absolute PSS-centered SOA and confidence judgments for correct and incorrect judgments, respectively. Thus, our results point to metacognitive monitoring of errors that is sensitive to the magnitude of SOAs. The results of Model 2 showed that although participants could monitor errors for their left-first judgments, interestingly, the corresponding relation was very weak but in the opposite direction for the right-first judgments errors. These results suggest a stronger metacognitive sensitivity for left-first temporal-order judgments. Critically, this result did not arise from a perception-based asymmetry across the response codes, as we did not find significant difference in accuracy proportions across response codes (i.e., participants’ accuracy was virtually the same for “left-first” and “right-first” responses). Thus, the observed asymmetry across response codes can be solely attributed to metacognitive asymmetry. In contrast, the raw data model revealed that participants could not significantly differentiate their correct and erroneous temporal order decisions. This result critically highlights that the metacognitive monitoring of the simultaneity perception is exclusive (and asymmetrical) for subjective asynchronies rather than objective ones.

Experiment 1 had several limitations that necessitated a replication study, particularly given the unexpected results of Model 2. Experiment 1 did not control for the visual angle, which could affect the simultaneity perception. We did not test all experimental SOAs with the same probability, where the extreme SOAs were tested less frequently than the middle SOAs. The final limitation is that while we equalized the right-left response conditions throughout the experiment, we did not equalize their probability across the SOAs. To test whether the results obtained in Experiment 1 are replicable after addressing these limitations, we conducted Experiment 2.

## Experiment 2

### Method

#### Participants

Thirty two undergraduate students from Koç University participated in Experiment 2 for half extra course credits. Two of them were discarded from the formal analyses as they were using psychiatric drugs. One was discarded as the R^2^ value of their psychometric function was below 0.8. As a result, twenty nine participants were included in the formal analyses (21 female, three left-handed, *M*_age_ = 20.76, *SD*_age_ = 1.79).

#### Materials and procedure

All materials and procedures were kept the same as in Experiment 1 except for the following procedural changes. Participants completed Experiment 2 on a chin rest ~ 34–35 cm away from the monitor to control for the visual angle across participants (~ 30°). All SOAs were tested for an equal number of trials in all testing blocks, and the side conditions were equalized across individual SOAs. Each SOA was tested for ten times. In five of them, the left disc appeared before the right. The experiment consisted of three test blocks, and each block consisted of 120 trials (i.e., 12 SOAs x 2 side conditions x 5 repeat in a test block). As a result, the experiment consisted of 360 trials in total (i.e., 120 trials x 3 test blocks). In addition, participants completed 24 practice trials before the experiment.

### Results

#### Response bias

As in Experiment 1, we tested whether there was an overall response or accuracy bias across the side responses. Parallel to Experiment 1, there were no such biases in either the accuracy or the side responses (side response: *t*(28) = 0.96, *p* = 0.35, Cohen’s d = 0.18; BF_10_ = 0.30, % error = 3.153e-4; where data are 3.33 (= 1/0.30) times more likely under the null than the alternative hypothesis; accuracy: *t*(28) = 0.73, *p* = 0.47, Cohen’s d = 0.14; BF_10_ = 0.25, % error = 3.205e-4; where data are 4 (= 1/0.25) times more likely under the null than the alternative hypothesis), revealing that the model results cannot be due to a potential bias in the side responses or differential overall accuracy.

#### Model 1

The slope for correct judgments as a function of absolute PSS-centered SOAs was significantly positive (ß_correct_ = 0.053, *SE* = 0.0029, 95% CI = [0.048 0.059], *p* < 0.001) while it was significantly negative for incorrect responses (ß_incorrect_ = -0.038, *SE* = 0.003, 95% CI = [-0.044 -0.031], *p* < 0.001). By transitivity, the slope difference was also statistically significant (ß_incorrect−correct_ = -0.091, *SE* = 0.004, 95% CI = [-0.099 -0.082], *p* < 0.001). For Model 1, results indicated higher confidence for incorrect responses (*M*_incorrect − correct_ = 0.11, *SE* = 0.016, 95% CI = [0.078 0.014], *p* < 0.001). These results corroborated the results of Experiment 1 (Fig. 2, left column).

#### Model 2

For Model 2, results point to an overall confidence bias for right first responses (*M*_right first − left first_ = 0.35, *SE* = 0.019, 95% CI = [0.31 0.39], *p* < 0.001 - Fig. 3, right column). The slope for the relationship between the PSS-centered SOA and confidence ratings was significantly positive for only the left-first judgments (ß_left first_ = 0.070, *SE* = 0.0016, 95% CI = [0.067 0.073], *p* < 0.001; ß_right first_ = 0.002, *SE* = 0.002, 95% CI = [-0.002 0.0066], *p* = 0.32), which was also significantly steeper than right-first judgments (ß_right first − left first_ = -0.068, *SE* = 0.0028, 95% CI = [-0.07 -0.06], *p* < 0.001). The second row of the right panel in Fig. 3 illustrates the relationship between subjective SOA and confidence judgements for response accuracy for Experiment 2.

#### Raw data - model

We also investigated our research question using a raw data, as in Experiment 1. The raw data-model revealed similar effects as in Experiment 1 (*M*_correct − incorrect_ = 0.35292, *SE* = 0.031584, 95% CI = [-0.41482 -0.29102], *p* < 0.00001; ß= 0.09318, *SE* = 0.003175, 95% CI = [0.08695 0.09940], *p* < 0.00001; simple slopes: ß_incorrect_ = 0.074, *SE* = 0.006,95% CI = [0.062 0.085], *p* < 0.001; ß_correct_ = 0.11, *SE* = 0.002, 95% CI = [0.11 0.12], *p* < 0.001), except that the slope difference between correct and incorrect responses was statistically significant (ß_correct − incorrect_ = 0.03818, *SE* = 0.006351, 95% CI = [-0.05063 -0.02573], *p* < 0.00001), pointing to a steeper slope for the correct responses. These findings replicate the results of Experiment 1.

### Interim discussion

The results of Model 1 and the raw data model fully replicated those of Experiment 1, whereas the results of Model 2 were partially consistent with those of Experiment 1. The very weak positive relationship between PSS-centered SOA and confidence ratings for right-first judgments in Experiment 1 was absent in Experiment 2. Experiment 3 was conducted to test the robustness of findings gathered in Experiment 2.

## Experiment 3: replication

### Method

#### Participants

Thirty one undergraduate students from Koç University participated in Experiment 3 for half extra course credit. Data of two participants were discarded from the formal analyses due to the use of antihistamines and epilepsy medicine. Data from four participants were discarded from the analyses due to their below 0.8 R^2^ value of the psychometric function fits. As a result, twenty five participants were included in the formal analyses (21 female, one left-handed, 1 using both hands, *M*_age_ = 20.60, *SD*_age_ = 1.35).

#### Materials and procedure

All the materials and procedures were identical to Experiment 2.

### Results

#### Response bias

We investigated the potential response or accuracy biases across the side responses as in the first two experiments. Paired sample t-test revealed no such biases for Experiment 3 (side response: *t(*24) = 0.49, *p* = 0.63, Cohen’s d = 0.098; BF_10_ = 0.24, % error = 2.599e-4; where data are 4.17 (= 1/0.24) times more likely under the null than the alternative hypothesis; accuracy: *t*(24) = 0.45, *p* = 0.66, Cohen’s d = 0.089; BF_10_ = 0.23, % error = 2.592e-4; where data are 4.35 (= 1/0.23) times more likely under the null than the alternative hypothesis), which is in line with the first two experiments.

#### Model 1

Results of Model 1 pointed to a significant positive slope for correct (ß_correct_ = 0.052, *SE* = 0.003, 95% CI = [0.046 0.058], *p* < 0.001) and a significant negative slope for incorrect judgments (ß_incorrect_ = -0.051, *SE* = 0.0034, 95% CI = [-0.058 -0.045], *p* < 0.001). By transitivity, the slope difference between the incorrect and correct judgments was also statistically significant (ß_incorrect−correct_ = -0.103, *SE* = 0.0046, 95% CI = [-0.11 -0.094], *p* < 0.001 - Fig. 3, left column). Model 1 also pointed to an overall high confidence for subjective incorrect responses (*M*_incorrect−correct_ = 0.096, *SE* = 0.017, 95% CI = [0.064 0.13], *p* < 0.001).

#### Model 2

Model 2 indicated higher confidence ratings for right-first judgments (*M*_left first − right first_ = -0.37, *SE* = 0.02, 95% CI = [-0.40 -0.33], *p* < 0.001). Furthermore, the slope that depicts the relationship between PSS-centered SOA and confidence ratings was significantly positive for both left-first and right-first judgments (ß_left first_ = 0.069, *SE* = 0.002, 95% CI = [0.066 0.072], *p* < 0.001; ß_right first_ = 0.0075, *SE* = 0.002, 95% CI = [0.003 0.012], *p* = 0.001) but it was steeper for left-first judgments (ß_left first − right first_ = 0.062, *SE* = 0.003, 95% CI = [0.056 0.067], *p* < 0.001 - Fig. 3, right panel).

#### Raw data - model

Finally, the raw data model revealed similar results as in the first two experiments (*M*_correct − incorrect_ = 0.267, *SE* = 0.032, 95% CI = [-0.33 -0.20], *p* < 0.00001; ß = 0.09484, *SE* = 0.0031, 95% CI = [0.089 0.10], *p* < 0.00001; simple slopes: ß_incorrect_ = 0.075, *SE* = 0.006, 95% CI = [0.06 0.09], *p* < 0.001; ß_correct_ = 0.11, *SE* = 0.002, 95% CI = [0.11 0.12], *p* < 0.001; ß_correct − incorrect_ = 0.039, *SE* = 0.0062, 95% CI = [-0.051 -0.027], *p* < 0.00001). These results replicate the first two experiments.

### Interim discussion

The results of Model 1 and raw data model fully replicated those of Experiments 1 and 2. Although the slope for the first responses on the left was significantly steeper than that of the first responses on the right, both slopes were significantly positive (Model 2). These results are partially consistent with Experiment 1, which revealed a significant negative slope, and Experiment 2, which did not reveal a significant slope for right-first judgments.

## Experiment 4: control experiment

In the first three experiments, the TOJ was evaluated based only on horizontally aligned stimuli. We conducted Experiment 4 to test whether the effects observed in the first three experiments could also apply to the vertically aligned stimuli. Thus, the aim of Experiment 4 was to control for the potential effect of stimuli alignment on the screen. If the effect we observed in the first three experiments is not specific to the alignment of the stimuli, the metacognitive asymmetry favoring one response option in the first three experiments should also be observed in Experiment 4, where the stimuli are vertically aligned on the screen.

### Method

#### Participants

Thirty nine Koç University undergraduate students participated in Experiment 4 in return for half extra course credit (32 female, six left-handed, *M*_age_ = 20.87, *SD*_age_ = 1.54). Data from all participants were included in the formal data analysis.

#### Materials and procedure

All the materials and procedures were identical to Experiment 2 and Experiment 3, except that the visual stimuli were vertically aligned. Thus, in Experiment 4, one of the two stimuli appeared either at the top or bottom of the screen. Participants pressed the keyboard’s up and down arrow keys to indicate “top” and “down first” responses, respectively.

### Results

#### Response bias

As in the first three experiments, we first investigated whether there was an overall bias in the accuracy favoring one type of side response (i.e., overall higher accuracy for “top first” or “bottom first” responses) or an overall bias in the side responses (i.e., overall higher rate of either “left” or “right first” responding). Two separate paired sample t-tests revealed response bias favoring “bottom first” response (*t*(37) = 2.63, *p* = 0.012, Cohen’s d = 0.43; BF_10_ = 0.29, % error = 6.798e-7; where data are 3.45 (= 1/0.29) times more likely under the null than the alternative hypothesis). Response accuracy percentage, however, favored “top first” responses (*t*(37) = -2.8, *p* = 0.0081, Cohen’s d = -0.45; BF_10_ = 0.20, % error = 4.129e-7; where data are 5 (= 1/0.20) times more likely under the null rather the alternative hypothesis).

#### Model 1

As in the case of first three experiments, although the relationship between the absolute PSS-centered SOA and confidence was significantly positive for correct judgments (ß_correct_ = 0.068, *SE* = 0.0025, 95% CI = [0.063 0.073], *p* < 0.001), this relation was significantly negative for the incorrect responses (ß_incorrect_ = -0.055, *SE* = 0.0027, 95% CI = [-0.060 -0.050], *p* < 0.001). By transitivity, the difference between the slopes was also statistically significant (ß_incorrect−correct_ = -0.12, *SE* = 0.0037, 95% CI = [ -0.12 -0.13], *p* < 0.001). The bottom row of the left panel in Fig. 3 illustrates the relationship between absolute PSS-centered SOA and confidence judgements separately for correct and incorrect judgments in Experiment 4 (SOM S4.1. for binned version). Also, as in the first three experiments, Model 1 also revealed that overall, participants reported higher confidence when their judgments were inaccurate (*M*_incorrect−correct_ = 0.080, *SE* = 0.013, 95% CI = [0.055 0.12], *p* < 0.001).

#### Model 2

For Model 2, results point to an overall confidence bias for top first responses (*M*_tot first − bottom first_ = 0.36, *SE* = 0.015, 95% CI = [0.33 0.39], *p* < 0.001 - Fig. 3, bottom row right column). The slope for the relationship between the PSS-centered SOA and confidence ratings was significantly positive for both the bottom-first judgments (ß_bottom first_ = 0.067, *SE* = 0.0013, 95% CI = [0.065 0.070], *p* < 0.001) and top-first judgments (ß_top first_ = 0.006, *SE* = 0.002, 95% CI = [0.002 0.010], *p* = 0.0022). The slope was significantly steeper for the bottom first judgments (ß_top first − bottom first_ = -0.061, *SE* = 0.0024, 95% CI = [-0.07 -0.06], *p* < 0.001). The fourth row of the right panel in Fig. 3 illustrates the relationship between subjective SOA and confidence judgements for response accuracy in Experiment 4.

#### Raw data - model

We also investigated our research question using the raw data, as in the first three experiments. The raw data model revealed similar effects as in the first three experiments (*M*_correct − incorrect_ = 0.46, *SE* = 0.026, 95% CI = [0.41 0.51], *p* < 0.00001; ß= 0.093, *SE* = 0.0027, 95% CI = [0.087 0.098], *p* < 0.00001; simple slopes: ß_incorrect_ = 0.083, *SE* = 0.0052, 95% CI = [0.073 0.093], *p* < 0.001; ß_correct_ = 0.10, *SE* = 0.0017, 95% CI = [0.099 0.11], *p* < 0.001), except that the slope difference between correct and incorrect responses was statistically significant (ß_correct − incorrect_ = 0.019, *SE* = 0.0055, 95% CI = [0.008 0.030], *p* = 0.0005), pointing to a steeper slope for the correct responses.

### Interim discussion

Overall, the results obtained in Model 1 and the raw data of Experiment 4 replicated the previous experiments. For both response types (i.e., “bottom first” and “top first” responses), the slopes were significantly positive. Furthermore, as in the first three experiments, Experiment 4 also revealed metacognitive asymmetry favoring “bottom first” responses, as reflected in a steeper slope for “bottom first” responses (Model 2). Together, these results show that the metacognitive asymmetry observed in the first three experiments was not dependent on the stimulus alignment.

## General discussion

The current study investigated whether human participants could keep track of their simultaneity perception as a function of subjective asynchronies.

In all four experiments, participants could correctly match their confidence judgements to the absolute magnitude of SOAs; confidence ratings for correct judgments increased with increasing absolute SOA, whereas they decreased for incorrect judgments. The sensitivity of simultaneity error judgments to the absolute magnitude SOA corroborates the earlier findings on temporal error monitoring (e.g., Öztel & Balcı, 2021; Öztel & Balcı, 2023a, 2024).

Crucially, the second model that tested the relationship between confidence ratings as a function of SOAs separately for left-first and right-first judgments showed in the first three experiments that simultaneity monitoring applies more prominently to left-first judgments. This asymmetry in the response persisted for Experiment 4, favoring “bottom first” response codes. Specifically, while the left-first (Experiment 1, 2, and 3) and bottom-first (Experiment 4) responses were monitored correctly, the right-first responses were either not monitored (Experiment 2; and top-first in Experiment 4) or incorrectly monitored (significant positive slope in Experiment 3). Note that the magnitude of the latter relation was very weak. Together, these results show that while errors in left-first/bottom-first judgments could be correctly monitored, this was not the case for the right-first/top-first judgments. Thus, simultaneity monitoring as a function of subjective asynchronies has an asymmetrical manifestation in favor of one response code.

One potential explanation for the consistent monitoring effect for only the left-first responses can be related to the linguistic asymmetries between time and space while describing them metaphorically (e.g., utilizing spatial metaphors to describe time more often than utilizing temporal metaphors to describe space). These asymmetries can eventually manifest themselves behaviorally in STEARC effect form, as asserted by Conceptual Metaphor Theory (CMT; e.g., “long duration”; where both “long” adjective has spatial characteristics; for a detailed discussion see Lakoff and Johnson 1999; Lakoff & Johnson, 2008; see also Winter et al., 2015). In behavioral forms, the STEARC effect manifests itself in faster responses/processing for left and right response mappings for “short duration” and “long duration”, respectively, while comparing durations of stimuli. This phenomenon, in return, simulates a horizontal mental timeline where earlier events appear on the left. Importantly, this representational asymmetry is alignment-independent such that it can be observed also for vertically aligned stimuli settings (“bottom-to-top” effect, Berraci et al., 2022).

A similar phenomenon might occur in the metacognitive processes such that tracking the direction of SOAs for the “left/bottom first” responses might be more intuitive than the “right/top first” responses due to their relative place in the mental timeline. Thus, the observed asymmetry might have derived from the potential mismatch between the mental timeline and the right stimulus to appear first. Accordingly, these results might be pointing to a metacognitive STEARC effect.

One critical finding we obtained is the discrepancy between the raw data model and its subjective counterpart (i.e., SOA-centered PSS; Model 1). As opposed to Model 1, the raw data model demonstrated no metacognitive monitoring of the simultaneity performance. This discrepancy between the results indicates that the phenomenological characteristics of the simultaneity monitoring rely only on the relative/subjective asynchronies rather than the objective ones. These phenomenological characteristics also differ from the temporal error monitoring ability that is investigated with the match between the reproduced durations and relative confidence and error directionality judgments as it relies both on the subjective/relative and objective/absolute timing errors (Öztel & Balcı, submitted). Taken together, the results of the current study put forth the phenomenological dissociations between metacognitive processing of simultaneity perception and temporal reproductions.

One way to investigate the metacognitive ability is by calculating meta-d’ based on the signal detection theoretic approach (Maniscalco & Lau, 2012). However, while this approach provides a bias-free parameterization of the metacognitive abilities in the categorical decision context (i.e., the performance can take only correct or incorrect forms), it falls short of capturing the metric characteristics of the errors made in temporal, spatial, and numerical (for a detailed discussion: Öztel & Balcı, 2024), which characterizes the metric error monitoring research compared to the categorical error monitoring. In the TOJ task, the performance mainly takes discrete correct-incorrect forms as in categorical decision tasks (e.g., 2-alternative forced choice tasks); however, the main objective of the current study was to investigate the metric characteristics of behavioral adjustments in the confidence judgements with respect to the relative direction of subjective asynchronies across different response codes and accuracy levels, which cannot be captured with meta-d’ approach.

## Conclusion

This study aimed to investigate the error monitoring dynamics for temporal order judgments. Overall, results pointed out that while the magnitude of SOAs is monitored through confidence ratings, the ability to monitor the simultaneity perception is asymmetrically manifested (i.e., only for left-first and bottom-first responses). This result, thus, points to a metacognitive STEARC effect.

## Electronic supplementary material

Below is the link to the electronic supplementary material.

Supplementary Material 1

## Data Availability

All data and codes used for this study are available in Open Science Framework (OSF) repository (https://osf.io/sw6cq/?view_only=105ad3a7fa844f7cbaacb55ae97ef066).

## Footnotes

1 Note that many perceptual judgments are also underlain by metric information (i.e., amount of evidence accumulated in favor of one hypothesis vs. another) but in these cases the metric information is not explicitly processed by the agents. Presumably, what agents have access to is the threshold-crossing of the accumulated evidence.
