# Supplementary material for: Asymmetrical monitoring of subjective asynchronies: a metacognitive generalized STEARC effect
Source: Psychol Res. 2025 Apr 28;89(3):96. doi: 10.1007/s00426-025-02123-2 (PMC12037655; doi:10.1007/s00426-025-02123-2)
Supplement: Supplementary file 1 — Supplementary Material 1 [file 426_2025_2123_MOESM1_ESM.docx]

**Supplementary Online Materials**

**S1.** Model 1

| **Exp Number** | | | | |
| --- | --- | --- | --- | --- |
|  | **Exp1** | **Exp2** | **Exp3** | **Exp 4** |
| AIC | 7859.99030 | 24674.38840 | 20619.87900 | 29719.81210 |
| BIC | 7935.64000 | 24748.14760 | 20692.68900 | 29796.97140 |
| LogLikel. | -3935.06525 | -12346.31358 | -10319.02957 | -14869.91464 |
| R-squared Marginal | 0.02424 | 0.04349 | 0.05580 | 0.07481 |
| R-squared Conditional | 0.12182 | 0.13731 | 0.12567 | 0.14789 |

**S1.1.** Model 1 fit results for four experiments

|  |  |  |  |  | **95% CI** | |  |  |  |
| --- | --- | --- | --- | --- | --- | --- | --- | --- | --- |
| **Exp Number** | **Names** | **Effect** | **Estimate** | **SE** | **Lower** | **Upper** | **df** | **t** | **p** |
| 1 | Intercept |  | 2.492039 | 0.055763 | 2.38275 | 2.60133 | 17.01 | 44.690 | < .00001 |
| 2 |  |  | 2.246280 | 0.048697 | 2.150836 | 2.34172 | 28.11 | 46.128 | < .00001 |
| 3 |  |  | 2.34975 | 0.043584 | 2.264329 | 2.435176 | 24.10 | 53.91278 | < .00001 |
| 4 |  |  | 2.404902 | 0.034510 | 2.337263 | 2.47254 | 37.32 | 69.687 | < .00001 |
| 1 | \|PSS-centered SOA\| |  | 0.008054 | 0.003685 | 8.327e-4 | 0.01528 | 3579.57 | 2.186 | 0.02888 |
| 2 |  |  | 0.007798 | 0.002128 | 0.003627 | 0.01197 | 10408.84 | 3.664 | 0.00025 |
| 3 |  |  | 6.378e-5 | 0.002242 | -0.004331 | 0.004458 | 8973.24 | 0.02845 | 0.97731 |
| 4 |  |  | 0.006518 | 0.001825 | 0.002942 | 0.01009 | 13640.59 | 3.572 | 0.00035 |
| 1 | Accuracy (subjective) | Incorrect - correct | 0.172298 | 0.024008 | 0.12524 | 0.21935 | 3580.10 | 7.177 | < .00001 |
| 2 |  |  | 0.109282 | 0.015969 | 0.077985 | 0.14058 | 10411.14 | 6.844 | < .00001 |
| 3 |  |  | 0.09609 | 0.016575 | 0.063608 | 0.128580 | 8976.99 | 5.79761 | < .00001 |
| 4 |  |  | 0.080159 | 0.013042 | -0.105721 | -0.05460 | 13645.32 | 6.146 | < .00001 |
| 1 | \|PSS-centered SOA\|* Accuracy (subjective) | ß✻ incorrect - correct | -0.047238 | 0.007879 | -0.062682 | -0.03179 | 3542.72 | -5.995 | < .00001 |
| 2 |  |  | -0.090688 | 0.004377 | -0.099267 | -0.08211 | 10430.73 | -20.719 | < .00001 |
| 3 |  |  | -0.10301 | 0.004608 | -0.112044 | -0.093979 | 8994.63 | -22.35351 | < .00001 |
| 4 |  |  | -0.122165 | 0.003716 | -0.12945 | -0.114882 | 13665.42 | -32.876 | < .00001 |

**S1.2.** Model 1 fixed effects parameter estimates for four experiments

| **Exp Number** | **Groups** | **Name** | **SD** | **Variance** | **Intraclass Correlation (ICC)** |
| --- | --- | --- | --- | --- | --- |
| 1 | participant | Intercept | 0.2310 | 0.05338 | 0.09455 |
| 2 |  |  | 0.2587 | 0.06692 | 0.09808 |
| 3 |  |  | 0.2140 | 0.04578 | 0.07400 |
| 4 |  |  | 0.2089 | 0.04364 | 0.07899 |
| 1 | Residual |  | 0.7150 | 0.51120 |  |
| 2 |  |  | 0.7845 | 0.61542 |  |
| 3 |  |  | 0.7569 | 0.57287 |  |
| 4 |  |  | 0.7133 | 0.50886 |  |

**S1.3.** Model 1 random components for four experiments

| **Exp Number** | **Moderator levels** |  |  | **95% CI** | |  |  |  | |
| --- | --- | --- | --- | --- | --- | --- | --- | --- | --- |
|  | **Accuracy (subjective)** | **Estimate** | **SE** | **Lower** | **Upper** | **Df** | **t** | | **p** |
| 1 | Incorrect | -0.01566 | 0.005662 | -0.02676 | -0.004563 | 3596 | -2.766 | 0.00570 | |
| 2 |  | -0.03755 | 0.003203 | -0.04383 | -0.03127 | 10424 | -11.72 | < .00001 | |
| 3 |  | -0.05144 | 0.003396 | -0.05810 | -0.04478 | 8990 | -15.15 | < .00001 | |
| 4 |  | -0.05456 | 0.002725 | -0.05991 | -0.04922 | 13658 | -20.02 | < .00001 | |
| 1 | correct | 0.03177 | 0.005135 | 0.02171 | 0.041839 | 3595 | 6.188 | < .00001 | |
| 2 |  | 0.05314 | 0.002894 | 0.04747 | 0.05881 | 10420 | 18.36 | < .00001 | |
| 3 |  | 0.05157 | 0.003023 | 0.04564 | 0.05750 | 8984 | 17.06 | < .00001 | |
| 4 |  | 0.06760 | 0.002477 | 0.06275 | 0.07246 | 13650 | 27.29 | < .00001 | |

**S1.4.** Model 1 simple slopes across four experiments

**S2.** Model 2

|  |  | **Exp Number** |  |  |
| --- | --- | --- | --- | --- |
|  | **1** | **2** | **3** | **4** |
| AIC | 7425.0344 | 23430.2552 | 19533.6247 | 28174.1322 |
| BIC | 7491.1023 | 23505.9220 | 19608.7395 | 28253.5171 |
| LogLikel. | -3720.9851 | -11725.2008 | -9777.0548 | -14098.1875 |
| R-squared Marginal | 0.1272 | 0.1383 | 0.1571 | 0.1665 |
| R-squared Conditional | 0.2146 | 0.2412 | 0.2149 | 0.2321 |

**S2.1.** Model 2 fit results for four experiments

|  |  |  |  |  | **95% CI** | |  |  |  |
| --- | --- | --- | --- | --- | --- | --- | --- | --- | --- |
| **Exp Number** | **Names** | **Effect** | **Estimate** | **SE** | **Lower** | **Upper** | **df** | **t** | **p** |
| 1 | Intercept |  | 2.30610 | 0.055089 | 2.19813 | 2.41408 | 18.33 | 41.86 | < .00001 |
| 2 |  |  | 2.14560 | 0.051352 | 2.04495 | 2.24625 | 28.69 | 41.78 | < .00001 |
| 3 |  |  | 2.27023 | 0.039777 | 2.19227 | 2.34819 | 25.00 | 57.07 | < .00001 |
| 4 |  |  | 2.35458 | 0.032876 | 2.29014 | 2.41901 | 38.82 | 71.62 | < .00001 |
| 1 | PSS-centered SOA |  | 0.03136 | 0.002346 | 0.02676 | 0.03596 | 3581.76 | 13.37 | < .00001 |
| 2 |  |  | 0.03624 | 0.001375 | 0.03354 | 0.03893 | 10411.12 | 26.36 | < .00001 |
| 3 |  |  | 0.03824 | 0.001410 | 0.03547 | 0.04100 | 8977.90 | 27.13 | < .00001 |
| 4 |  |  | 0.03662 | 0.001174 | 0.03432 | 0.03892 | 13643.84 | 31.19 | < .00001 |
| 1 | Response | right first - left first | 0.40153 | 0.030979 | 0.34081 | 0.46224 | 3584.45 | 12.96 | < .00001 |
| 2 |  |  | 0.34867 | 0.018520 | 0.31237 | 0.38497 | 10415.36 | 18.83 | < .00001 |
| 3 |  |  | 0.36707 | 0.018962 | -0.40424 | -0.32991 | 8986.12 | -19.36 | < .00001 |
| 4 |  | Top first – bottom first | 0.36199 | 0.015418 | 0.33177 | 0.39221 | 13648.73 | 23.48 | < .00001 |
| 1 | PSS-centered SOA* Response | ß✻ right first - left first | -0.08181 | 0.004728 | -0.09107 | -0.07254 | 3585.89 | -17.30 | < .00001 |
| 2 |  |  | -0.06802 | 0.002751 | -0.07341 | -0.06263 | 10411.58 | -24.72 | < .00001 |
| 3 |  |  | -0.06146 | 0.002837 | 0.05590 | 0.06702 | 8984.46 | 21.66 | < .00001 |
| 4 |  | ß✻ top first - bottom first | -0.06103 | 0.002357 | -0.06565 | -0.05641 | 13649.70 | -25.89 | < .00001 |

**S2.2.** Model 2 fixed effects parameter estimates for four experiments

| **Exp Number** | **Groups** | **Name** | **SD** | **Variance** | **Intraclass Correlation (ICC)** |
| --- | --- | --- | --- | --- | --- |
| 1 | participant | Intercept | 0.2244 | 0.05034 | 0.1001 |
| 2 |  |  | 0.2721 | 0.07403 | 0.1194 |
| 3 |  |  | 0.1933 | 0.03735 | 0.06850 |
| 4 |  |  | 0.1970 | 0.03883 | 0.07870 |
| 1 | Residual |  | 0.6726 | 0.45234 |  |
| 2 |  |  | 0.7389 | 0.54595 |  |
| 3 |  |  | 0.7126 | 0.50785 |  |
| 4 |  |  | 0.6742 | 0.45450 |  |

**S2.3.** Model 2 random components for four experiments

| **Exp Number** | | **Moderator levels** |  |  | **95% CI** | |  |  |  |
| --- | --- | --- | --- | --- | --- | --- | --- | --- | --- |
|  | **Response** | | **Estimate** | **SE** | **Lower** | **Upper** | **Df** | **t** | **p** |
| 1 | | Left first | 0.072266 | 0.003090 | 0.06621 | 0.078323 | 3584 | 23.390 | < .00001 |
| 2 | |  | 0.070245 | 0.001619 | 0.067071 | 0.073420 | 10411 | 43.377 | < .00001 |
| 3 | |  | 0.068965 | 0.001649 | 0.065733 | 0.07220 | 8981 | 41.830 | < .00001 |
| 4 | | Bottom first | 0.067131 | 0.001246 | 0.064689 | 0.06957 | 13646 | 53.893 | < .00001 |
| 1 | | Right first | -0.009540 | 0.003555 | -0.01651 | -0.002569 | 3584 | -2.683 | 0.00732 |
| 2 | |  | 0.002227 | 0.002223 | -0.002131 | 0.006585 | 10412 | 1.002 | 0.31651 |
| 3 | |  | 0.007509 | 0.002298 | 0.003005 | 0.01201 | 8982 | 3.268 | 0.00109 |
| 4 | | Top first | 0.006104 | 0.001996 | 0.002182 | 0.01002 | 13647 | 3.059 | 0.0223 |

**S2.4.** Model 2 simple slopes across four experiments

**S3.** Raw data-model

|  |  | **Exp Number** |  |  |
| --- | --- | --- | --- | --- |
|  | **Exp1** | **Exp2** | **Exp3** | **Exp4** |
| AIC | 6881.6731 | 20755.6980 | 17274.0188 | 24700.4919 |
| BIC | 6944.1410 | 20828.5634 | 17346.3134 | 24776.9788 |
| LogLikel. | -3447.5044 | -10386.5215 | -8645.8418 | -12359.9183 |
| R-squared Marginal | 0.2381 | 0.3123 | 0.3298 | 0.3404 |
| R-squared Conditional | 0.3205 | 0.4078 | 0.3905 | 0.4009 |

**S3.1.** Raw data-model fit results for four experiments

|  |  |  |  |  | **95% CI** | |  |  |  |
| --- | --- | --- | --- | --- | --- | --- | --- | --- | --- |
| **Exp Number** | **Names** | **Effect** | **Estimate** | **SE** | **Lower** | **Upper** | **df** | **t** | **p** |
| 1 | (Intercept) |  | 1.65761 | 0.061181 | 1.53770 | 1.777523 | 31.93 | 27.093 | < .00001 |
| 2 |  |  | 1.45971 | 0.050954 | 1.35984 | 1.55958 | 33.09 | 28.648 | < .00001 |
| 3 |  |  | 1.58889 | 0.042775 | 1.50505 | 1.67273 | 30.74 | 37.145 | < .00001 |
| 4 |  |  | 2.25567 | 0.032556 | 2.191866 | 2.31948 | 44.85 | 69.287 | < .00001 |
| 1 | SOA |  | 0.09856 | 0.006144 | 0.08652 | 0.110605 | 3581.68 | 16.042 | < .00001 |
| 2 |  |  | 0.09318 | 0.003175 | 0.08695 | 0.09940 | 10411.01 | 29.346 | < .00001 |
| 3 |  |  | 0.09484 | 0.003122 | 0.08873 | 0.10096 | 8979.00 | 30.379 | < .00001 |
| 4 |  |  | 0.09261 | 0.002729 | 0.087256 | 0.09796 | 13646.47 | 33.928 | < .00001 |
| 1 | Accuracy (objective) | incorrect - correct | -0.44249 | 0.067161 | -0.57412 | -0.310858 | 3581.21 | -6.589 | < .00001 |
| 2 |  |  | -0.35292 | 0.031584 | -0.41482 | -0.29102 | 10409.49 | -11.174 | < .00001 |
| 3 |  |  | -0.26686 | 0.032100 | -0.32977 | -0.20394 | 8974.01 | -8.313 | < .00001 |
| 4 |  |  | -0.58627 | 0.022288 | 0.542585 | 0.62995 | 13655.38 | -26.304 | < .00001 |
| 1 | SOA*Accuracy (objective) | ß*incorrect-correct | -0.01444 | 0.012288 | -0.03852 | 0.009649 | 3581.66 | -1.175 | 0.24 |
| 2 |  |  | -0.03818 | 0.00635 | -0.05063 | -0.02573 | 10411.04 | -6.012 | < .00001 |
| 3 |  |  | -0.03888 | 0.006238 | -0.05111 | -0.02665 | 8978.17 | -6.233 | < .00001 |
| 4 |  |  | -0.01903 | 0.005456 | -0.02972 | -0.008337 | 13645.65 | -3.488 | 0.00049 |

**S3.2.** Raw data-model fixed effects parameter estimates for four experiments

| **Exp Number** | **Groups** | **Name** | **SD** | **Variance** | **Intraclass Correlation (ICC)** |
| --- | --- | --- | --- | --- | --- |
| 1 | participant | Intercept | 0.2172 | 0.04719 | 0.1082 |
| 2 |  |  | 0.2609 | 0.06808 | 0.1388 |
| 3 |  |  | 0.1983 | 0.03932 | 0.09058 |
| 4 |  |  | 0.1887 | 0.03559 | 0.09172 |
| 1 | Residual |  | 0.6235 | 0.38881 |  |
| 2 |  |  | 0.6499 | 0.42237 |  |
| 3 |  |  | 0.6283 | 0.39477 |  |
| 4 |  |  | 0.5937 | 0.35242 |  |

**S3.3.** Raw data-model random components for four experiments

| **Exp Number** | **Moderator levels** |  |  | **95% CI** | |  |  |  |
| --- | --- | --- | --- | --- | --- | --- | --- | --- |
|  | **Accuracy (objective)** | **Estimate** | **SE** | **Lower** | **Upper** | **Df** | **t** | **p** |
| 1 | Incorrect | 0.09134 | 0.011508 | 0.06878 | 0.1139 | 3582 | 7.937 | < .00001 |
| 2 |  | 0.07409 | 0.005997 | 0.06233 | 0.08584 | 10411 | 12.35 | < .00001 |
| 3 |  | 0.07540 | 0.005841 | 0.06395 | 0.08685 | 8979 | 12.91 | < .00001 |
| 4 |  | 0.08309 | 0.005199 | 0.07290 | 0.09328 | 13647 | 15.98 | < .00001 |
| 1 | correct | 0.10578 | 0.004308 | 0.09733 | 0.1142 | 3580 | 24.553 | < .00001 |
| 2 |  | 0.11227 | 0.002090 | 0.10817 | 0.11636 | 10408 | 53.71 | < .00001 |
| 3 |  | 0.11429 | 0.002200 | 0.10997 | 0.11860 | 8973 | 51.96 | < .00001 |
| 4 |  | 0.10212 | 0.001659 | 0.09887 | 0.10537 | 13640 | 61.55 | < .00001 |

**S3.4.** Raw data-model simple slopes across three experiments

**
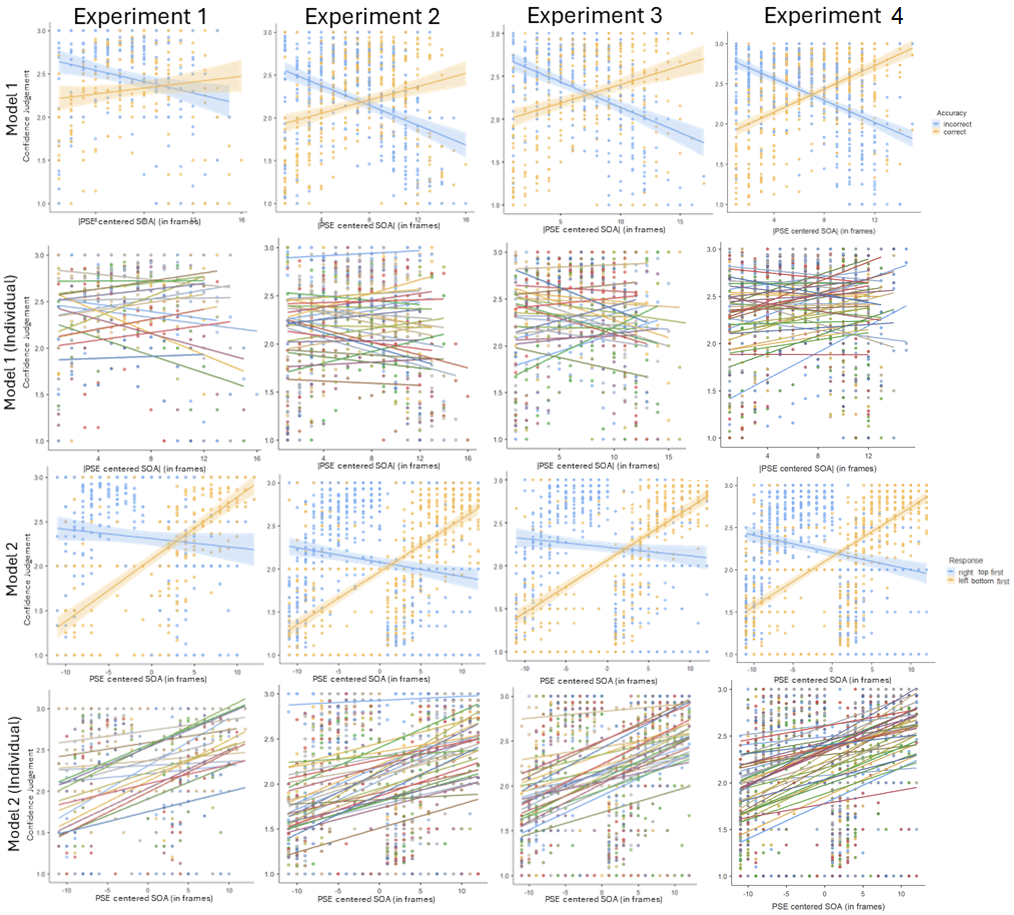
**

**S4.1.** The behavioral patterns for binned confidence rating across different SOAs for Model 1 and Model 2. The individual graphs (row 2 and row 4) depict individual average slopes for each participant. The sign of the values in the x axis for Model 2 depict the stimulus first appearing direction (negative for “right first”, positive for “left first”)


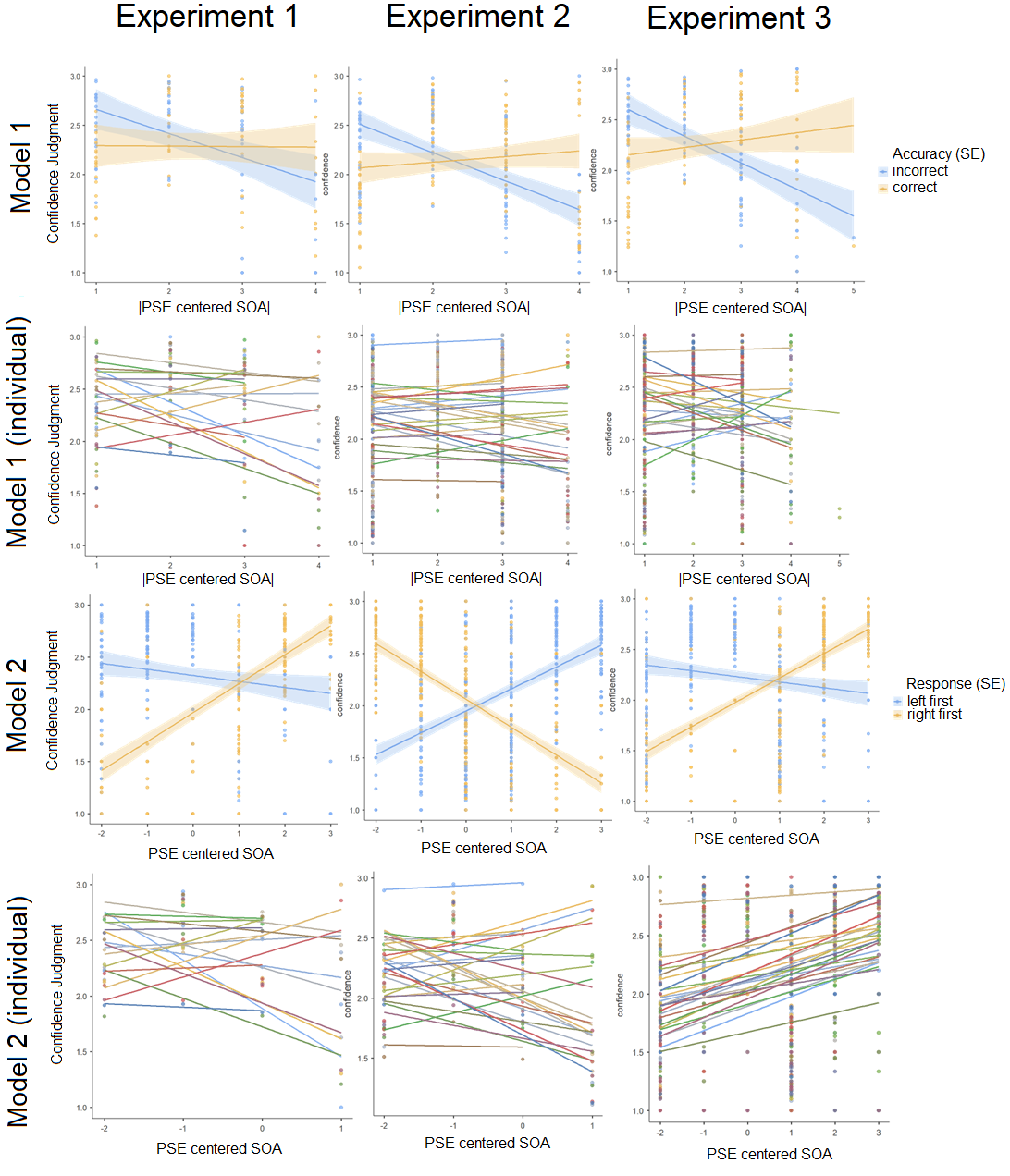


**S4.2.** Figure S4.1. with larger bins
